# How Do Adolescent Smoking Prevention Interventions Work in Different Contextual Settings? A Qualitative Comparative Study Between the UK and Colombia

**DOI:** 10.1007/s12529-023-10211-z

**Published:** 2023-09-11

**Authors:** Sharon Sánchez-Franco, Shannon C. Montgomery, Erika S. Torres-Narvaez, Ana M. Ramírez, Jennifer M. Murray, Christopher Tate, Blanca Llorente, Linda Bauld, Ruth F. Hunter, Frank Kee, Olga L. Sarmiento

**Affiliations:** 1 School of Medicine, Universidad de los Andes, Carrera 1 # 18A-10 Block Q, 111711018 Bogotá, Colombia; 2 College of Human Sciences, Florida State University, Florida, USA; 3 Centre for Public Health, School of Medicine, Dentistry and Biomedical Sciences, Queen’s University Belfast, Belfast, Northern Ireland, UK; 4 Fundación Anaás, Bogotá, Colombia; 5 College of Medicine and Veterinary Medicine, Usher Institute and SPECTRUM Consortium, University of Edinburgh, Edinburgh, Scotland, UK

**Keywords:** Smoking prevention, Qualitative analysis, Behavior change, Health interventions, Adolescent health

## Abstract

**Background:**

Adolescent smoking is associated with significant health and social risks. Previous research has demonstrated the effectiveness of interventions based on behavior change theories in preventing adolescent smoking uptake. However, evidence from the theory-based perspective of evaluation is limited, especially for how such complex interventions work, and how they work when implemented in different contextual settings.

**Method:**

A comparative qualitative analysis was conducted to explore various influences on behavior change among participants taking part in two smoking prevention interventions in Northern Ireland and Bogotá. Twenty-seven focus groups were conducted in 12 schools (6 in Northern Ireland and 6 in Bogota, *n* = 195 pupils participated; aged 11–15 years). The Theoretical Domains Framework guided a content analysis of the data.

**Results:**

We found similarities across settings in terms of knowledge, skills, and beliefs related to smoking or vaping behavior change, as well as differences in contextual resources and social influence. Different environmental resources included availability to purchase tobacco products in the neighborhoods and previous information about tobacco risk. Participants in both interventions perceived behavioral change outcomes related to personal skills and intention to not smoke or vape.

**Conclusion:**

These findings have highlighted how both individual factors and contextual resources influence behavior change for smoking prevention in practice. Local contextual factors and social influences affecting pupils should be taken into account in the implementation and evaluation of health behavior change interventions. In particular, this study supports using social and contextual influence strategies in interventions to reduce the onset of adolescent smoking and vaping.

**Supplementary Information:**

The online version contains supplementary material available at 10.1007/s12529-023-10211-z.

## Introduction

Globally, adolescent smoking is associated with long-term tobacco use, risky behaviors, and non-communicable diseases [1]. About 90% of smokers begin smoking before they are 19 years old and 43.8 million children aged 13–15 years use tobacco products worldwide [2, 3].

Smoking is considered to be a “socially contagious behavior” and adolescents are particularly susceptible to social influences [4–6]. A wide range of environmental factors increase susceptibility for smoking behavior among adolescents [7, 8]. Current global tobacco control strategies are focused on addressing the effect of emergent nicotine products on adolescent tobacco use [9]. Appeals such as the availability of different flavors for e-cigarettes, social media advertising, and social influences increase adolescents’ exposure to tobacco and, consequently, their smoking behavior [10–12].

Different strategies aimed at eliciting social influences are supported by existing theories of behavior change that integrate interpersonal and environmental factors [13]. Studies have assessed the effectiveness of interventions based on a variety of behavioral change theories to prevent or reduce adolescent smoking [14–17]. However, the effectiveness perspective of evaluation would not provide sufficient evidence for intervention implementation [18]. A more theory-based perspective on evaluation focuses on understanding the mechanisms of interventions, *how* an intervention works, and how this may vary across different settings and individuals [18, 19]. The mechanisms by which these types of interventions may change participants’ behavior are unclear [20], and the pathways at the social network and norms levels are rarely explored [21]. Therefore, understanding how such interventions work, for whom, and why is fundamental to evaluating their implementation and supporting real-world research and decision-making on public health [18].

The MECHANISMS study aims to better characterize the potential mechanisms of action of two smoking prevention interventions in schools (ASSIST and Dead Cool) in two different contextual settings [22]. Both smoking prevention interventions have been shown to be effective in the United Kingdom (UK) for reducing the number of adolescents that initiate smoking [23, 24]. Since both interventions incorporate social influence using different behavioral strategies, the mechanisms by which behavior change occurs may be different in the two contrasting settings. This study compares the interventions in a high-income setting and a middle-income setting using data obtained in Northern Ireland (UK) and Bogotá (Colombia).

Research on the mechanisms of interventions increases understanding of how behavioral changes are achieved, and how these changes may vary across different contexts and groups of participants [18]. Behavior change interventions are typically embedded in complex social systems. Therefore, we expect that the same program will have varying resources, functions, and outcomes across multiple contextual boundaries [25]. Theory-based evaluation of interventions focuses on assessing the interplay of the context on intervention outcomes and facilitates the integration of conceptual and empirical data [18].

The ASSIST and Dead Cool interventions aim to prevent the onset of adolescent smoking in schools, but are based on different behavior change theories. The intervention logic models have been previously published in the MECHANISMS study protocol [22]. The ASSIST intervention is based on the Diffusion of Innovations Theory, which outlines the process by which a novel behavior is spread among the members of a community in order to incorporate the new behavior into the social system [26]. In the intervention, a group of nominated “peer supporters” are trained to understand the dangers and risks of smoking and are subsequently asked to encourage their school peers not to smoke through informal conversations in everyday situations [24]. Therefore, the intervention design explicitly leverages adolescent friendship networks. The Dead Cool intervention is delivered through a more conventional classroom-based pedagogy and is based on the Theory of Planned Behavior, which outlines the process by which individual factors are targeted to develop a behavioral intention, the major determinant of behavior [27]. During the intervention, participants examine their skills and the influences on smoking behavior [23].

The Theoretical Domains Framework (TDF) describes an integrative process for synthesizing the theories and constructs related to behavior change to make them more accessible for various disciplines, and to assist the application of theory in the design and evaluation of interventions [28]. The TDF includes 14 conceptual domains to explain behavior change, and its structure has been validated and updated using conceptual consensus and global empirical data about its applicability [28]. The TDF has been applied in the literature to assess interventions that involve health professionals, patients, and the public population. This literature demonstrates that it is useful to optimize qualitative research to enquire about behavioral determinants [29].

Quantitative analytical techniques have enabled researchers to empirically examine the individual and contextual characteristics of interventions, including those related to the social environment and social norms, which are related to adolescent smoking behavior. In particular, previous research has demonstrated that the ASSIST and Dead Cool interventions effectively reduce the likelihood of adolescents initiating smoking in the UK [23, 24], and how social network structures influenced the changes in social norms for smoking [6]. However, qualitative research methods can explore a wider range of influences for behavioral change, beyond those which can be discerned through quantitative methods alone [30]. Qualitative research can reveal specific local interplays invoking individual agency, which is imperative for understanding behavioral change [30]. Emergent studies have used qualitative techniques to assess health behavior changes during behavioral interventions [31–33]. However, there is limited literature on qualitative research exploring the mechanisms of complex health behavior interventions [34]. Qualitative inquiry has the potential to reveal connections between smoking behavior and interventions, while maintaining a focus on the context of the implementation.

The current study aims to compare the influences on the ASSIST and Dead Cool participants’ behavioral change related to smoking or vaping in two different contexts (Bogotá and Northern Ireland). The TDF was used to conduct a theory-based analysis of the behavioral influences involved in the interventions in different contexts using comparable conceptual constructs. We expected the results of this analysis to inform how the interventions worked in the different settings in terms of the mechanisms of action of the smoking prevention interventions.

### Study Settings

Bogotá and Northern Ireland provide contrasting contexts that can illuminate underpinning mechanisms of smoking prevention interventions, including the varying social norms, cultures, policies, socioeconomic standing, and smoking behaviors. Bogotá is the capital city of Colombia, a Spanish-speaking country in Latin America, and has over 7.2 million inhabitants. Northern Ireland is part of the UK, an English-speaking country with approximately 2 million inhabitants [35].

Social differences between the countries are apparent in terms of population composition, economic standing, and available resources. For example, Colombia has an absolute poverty rate of 4.1% while in the UK it is 0.2% [36]. In addition, in Colombia, there are 26 pupils per teaching staff in secondary public institutions, and only 17 in the UK [36].

Both countries have partially fulfilled their commitments under the WHO Framework Convention on Tobacco Control (FCTC). The World Health Organization (WHO) has recognized the UK policies to monitor the tobacco epidemic, create smoke-free environments, include health warning labels, implement anti-tobacco mass media campaigns, and raise taxes on tobacco. Colombia has achieved high levels of implementation of smoke-free environments and regulations to enforce bans on tobacco advertising, promotion, and sponsorship [9]. The Global Tobacco Industry Interference Index is higher in Colombia (ranking 76) than in the UK (ranking 32), which indicates the tobacco industry’s attempts to limits the countries efforts to regulate and implement the control tobacco policies [37].

Sub-national implementation of the FCTC contrasts with the current state of the tobacco epidemic. The prevalence of tobacco use among adolescents is higher in Bogotá than in Northern Ireland. In Bogotá, 10.6% of adolescents aged 13–15 years use conventional cigarettes, and 11.2% use e-cigarettes [38]. By contrast, in Northern Ireland, 4% of adolescents aged 11–16 years use conventional cigarettes, and 3% use e-cigarettes [39].

Implementation of school-based smoking prevention programs is also different in both settings. In Northern Ireland, pupils are provided with smoking prevention information as part of the school curriculum from an early age [40]. Conversely, in Bogotá, tobacco education is a suggested (not compulsory) component of the school curriculum [41], and 51% of students (13–15 years) reported having received information about the health risks of tobacco in school [38].

## Methods

This research formed part of the MECHANISMS study, a concurrent nested mixed-methods study [22]. Here, we present findings from the qualitative component, a descriptive case study using data collected through focus group discussions. Electronic Supplementary Material 1 shows the Consolidated Criteria for Reporting Qualitative Research (COREQ checklist).

### Participants

The MECHANISMS study included 1315 participants aged 11–15 years old in Northern Ireland (*N* = 677) and Bogotá (*N* = 638) who received either the ASSIST or Dead Cool interventions, and were active in the study. Using a whole school year approach, ASSIST was delivered in three schools in Northern Ireland (*N* = 393 students) and three schools in Bogotá (*N* = 333 students), meanwhile Dead Cool was delivered in three schools in Northern Ireland (*N* = 284 students) and three schools in Bogotá (*N* = 305 students).

Out of the 726 students that received the ASSIST intervention, 75 students were recruited to participate in the focus groups (Northern Ireland = 59; Bogotá = 16). Overall, 142 students were trained as peer supporters (75 in Northern Ireland; 67 in Bogotá) and 41 were selected to participate in the focus groups at the end of the intervention (Northern Ireland = 25; Bogotá = 16). Out of the 589 students that received the Dead Cool intervention, 79 were recruited to participate in the focus groups at the end of the intervention (Northern Ireland = 55; Bogotá = 24).

The team followed the logic of maximum variation sampling to guide the recruitment process up to data saturation aiming to ensure the inclusion of participants with varying characteristics in both interventions, including smokers and non-smokers, girls and boys, and students from different school classes. Participants were invited during the data collection sessions, and interested students (and their parents) completed written consent forms before the focus groups. In Bogotá, participants were recruited face-to-face in the classroom. In Northern Ireland, participants were recruited using an invitation sheet in the school.

Ethical approval was granted from the School of Medicine, Dentistry and Biomedical Sciences Ethics Committee at Queens University Belfast in Northern Ireland (reference number 18.43; v3 Sept 2018), and the Research Ethics Committee of the Universidad de los Andes in Bogotá (reference number 937—July 30, 2018).

### Procedure and Materials

Both interventions were delivered in the schools according to their respective intervention protocol. In Bogotá, both interventions were culturally adapted before the implementation, including a fidelity assessment [42]. As part of the MECHANISMS study, a sociodemographic questionnaire was completed by all students who received the smoking prevention interventions [43] (see Electronic Supplementary Material 2 which details their sociodemographic characteristics). At the end of the interventions, focus groups were conducted with participants using a semi-structured topic guide. Questions covered domains of behavior change related to smoking and participants’ experience with the interventions according to their role including pupils or peer supporters (see Electronic Supplementary Material 3).

In total, 17 focus groups were conducted with the ASSIST participants (Northern Ireland = 9; Bogotá = 8), and 10 focus groups were conducted with the Dead Cool participants (Northern Ireland = 4; Bogotá = 6). All focus groups were facilitated in the schools by a trained interviewer using English or Spanish according to the participant’s native language. The focus groups were audio-recorded with the prior permission of all participants. Then, the recordings were anonymized and transcribed verbatim in their original language.

### Data Analysis

A deductive content analysis was used to compare the influences on the ASSIST and Dead Cool participants’ behavior change related to smoking in both Northern Ireland and Bogotá [44]. The focus groups were encoded in the original language by three bilingual and independent coders.

The research team used two cycles of coding. The first coding cycle identified the behavior change constructs related to the interventions using the TDF domains [28]. It included 14 categories corresponding to the TDF conceptual domains: knowledge, skills, social role, beliefs about capabilities, optimism, beliefs about consequences, reinforcement, intentions, goals, decision processes, environmental context and resources, social influence, emotion, and behavioral regulation. The second coding cycle disaggregated sub-categories, including separated sub-categories related to vaping behavior when the data were explicit. A total of 24 sub-categories were identified. Also, empty TDF domains were suppressed including optimism, reinforcement, goals, decision processes, emotion, and behavioral regulation. In addition, an emergent category was included that described the perceived behavior changes as intervention outcomes. The final codebook included the following categories: knowledge, skills, social role for health promotion, beliefs about capabilities, beliefs about consequences, intentions, social influences, environmental context and resources, and perceived behavior changes.

To establish inter-coder reliability, the coding team employed debriefing and member checking at the end of each coding cycle. After codification, we compared the content of the categories using axial matrices including setting (Bogotá vs. Northern Ireland) and intervention (Dead Cool vs. ASSIST). NVivo qualitative data analysis software was used (QSR International 193 Pty Ltd. Version 12 Pro). In addition, a descriptive analysis of the sociodemographic characteristics of the students who received the smoking prevention interventions was conducted using the statistical package Stata (StataCorp, 2015; Stata Statistical Software: Release 14; College Station, TX: StataCorp LP).

## Results

Electronic Supplementary Material 2 highlights the sociodemographic characteristics of the students who received the smoking prevention interventions. Participants had similar socioeconomic characteristics overall, with the majority of students being categorized in low and middle socioeconomic categories in both settings. A smaller proportion of the students in the Bogotá sample lived with both parents. In addition, students had some varying characteristics derived from contextual differences. For example, Bogotá had some participants who were slightly older (15 years old and older) and more ethnic minority students than Northern Ireland.

It was identified the 9 hierarchical categories that are listed by highest to lowest saturation (number of times that a theme was mentioned by the participants) in Electronic Supplementary Material 4. The meaning of each hierarchical category corresponds to:

- Knowledge: Awareness of tobacco use and associated products, including knowledge of cigarettes, e-cigarettes, and smoking behavior and gaps in knowledge.
- Skills: Interpersonal skills relevant to the interventions acquired through practice, including communication skills and refusal skills.
- Social role for health promotion: Experiences and opinions regarding the student’s role in encouraging others not to smoke, through conversations about the risk of smoking. It includes encouraging peers and family members.
- Beliefs about capabilities: Perceived confidence about one’s own ability or talent in relation to the prevention of smoking behavior. It includes perceived competence to encourage others not to smoke and self-efficacy to refuse.
- Beliefs about consequences: Recognition of the possible outcomes or consequences of smoking behavior. It includes health and social risk perception, and perception of benefits.
- Intentions: Conscious decision of wanting (or not wanting) to smoke or vape. It includes intentions to smoke, intentions to vape, or intentions to not smoke or vape.
- Social influences: Interpersonal processes that are associated with the changing of thoughts, feelings, or behaviors about smoking, such as social pressure and social norms. It includes family and peer influences, descriptive social norms, and social acceptance.
- Environmental context and resources: elements of the student’s environment that act as barriers or facilitators for smoking behavior, including resources in families, schools, and neighborhoods. In addition, we identified availability of tobacco products and exposure to tobacco-related advertising.
- Perceived behavior changes: changes in beliefs, knowledge, skills, behaviors, attitudes, or other constructs that participants report as an outcome of the interventions.

Table 1 presents the comparison of categories obtained in the ASSIST and Dead Cool intervention schools as well as in Bogotá and Northern Ireland. We identified similarities and differences between settings and interventions that are summarized in four main results: (i) knowledge, skills, and beliefs operate in similar way; (ii) social influences are similar but operate in a different way across settings; (iii) adolescents have very different environmental context and resources across settings; and (iv) perceived behavior changes outcomes were different across interventions and settings. Details of each main result are described below.

**Table 1:** Identified content of behavioral change domains related to smoking and vaping, and perceived intervention outcomes in Bogotá and Northern Ireland

**Content of domains**	**Setting/intervention**			
	**Bogotá**		**N. Ireland**	
	**A**	**DC**	**A**	**DC**
**Environmental context and resources**				
Availability to purchase cigarettes in neighborhood	x	x		
Adults purchase cigarettes for adolescents			x	x
Smokers in families	x	x	x	x
Smokers at school	x	x		
Smokers in neighborhood	x	x	x	x
E-cigarette advertising in social media	x	x	x	x
Availability to purchase e-cigarettes online			x	x
Consumption of other substances in neighborhood	x	x		
Anti-smoking information in school and neighborhood			x	x
**Knowledge**				
Knowledge about consequences	x	x	x	x
Knowledge about ingredients	x		x	
Knowledge about e-cigarettes			x	x
Request for more information about e-cigarettes	x	x	x	
Request for more information about other substances	x	x		
**Skills**				
Communications skills	x		x	
Personal skills for freedom of expression	x			
Personal skills for empathy	x			
Personal skills to refuse		x		x
Personal skills for awareness of advertising		x		x
**Social role for health promotion**				
Encourage other peers to not smoke	x		x	
Encourage friends to not smoke		x		x
Encourage family members to not smoke	x		x	x
**Beliefs about capabilities**				
Self-efficacy to not smoke	x	x	x	x
**Beliefs about consequences**				
Perception of health consequences	x	x	x	x
Perception of social consequences related to the family	x		x	x
Perception of social consequences related to peers			x	x
**Intentions**				
Intention to not smoke	x	x	x	x
Previous smoking or vaping due to curiosity and social pressure	x	x		
Previous smoking or vaping for stress relief			x	
**Social influences**				
Beliefs that many other peers smoke	x	x		
Beliefs that few other peers smoke			x	x
Beliefs that many other family members smoke	x	x	x	x
Beliefs that e-cigarettes are more approved of			x	x
Peer pressure and social acceptance to smoke	x	x		
Peer pressure to fit in			x	x
Family do not approve of smoking behavior	x	x	x	x
**Perceived change in behavior**				
Improved intention to not smoke		x		x
Maintained intention to not smoke				x
Improved knowledge about consequences and ingredients	x	x	x	x
Improved beliefs about health consequences	x	x	x	x
Improved beliefs about social consequences				x
Improved personal skills for empathy	x			
Improved personal skills to refuse		x		x
Improved awareness of social influence		x		x
Improved awareness of tobacco advertising		x		x
Improved encouragement of other peers to not smoke	x		x	

Content corresponds to the qualitative analysis of the structural categories. To see the resulting sub-categories, visit Electronic supplementary material 3

*A* ASSIST intervention, *DC* Dead Cool intervention

### Knowledge, Skills, and Beliefs Operate in a Similar Way

We identified similarities in knowledge, skills, and beliefs across the interventions in both settings. ASSIST participants mentioned knowledge about components of the cigarettes, communication skills, and encouraging other peers and family members to not smoke. It is important to note that only peer supporters, who were trained, mentioned personal skills and the social role for health promotion. Meanwhile, Dead Cool participants mentioned development of personal skills to refuse offers of cigarettes and increased awareness of advertising. In both programs and both settings, participants mentioned knowledge and perceptions about health consequences, self-efficacy to not smoke or vape, intentions to not smoke or vape, previous experiences using tobacco, encouraging friends to not smoke, and social influences for smoking.

In addition, differences across settings were identified. In Bogotá, all groups reported previous smoking and/or vaping due to curiosity and social pressure. For example, one pupil mentioned: “Well, it has been mostly with e-cigarettes. The first time, I did it out of curiosity and the second time for the flavor, because I liked it” (Dead Cool pupil, 1st October 2019, Bogotá). In addition, Bogotá’s pupils wanted more information about other substances and peer supporters mentioned personal skills for freedom of expression and empathy. By contrast, in Northern Ireland, participants reported previous smoking and vaping experiences for stress relief. For example, one participant mentioned: “Normally, if you’re stressing out you just light up a fag [cigarette]. It makes everything 10 times better, it’s just like a big relief” (ASSIST pupil, 19th June 2019, Northern Ireland). In addition, Northern Irish pupils requested more information about e-cigarettes and mentioned perceptions of peers and social consequences and their awareness of social influences around smoking.

### Social Influences Are Similar But Operate in a Different Way

In both settings, participants mentioned that families and peers are important influences for smoking behavior. However, the social influence for smoking and vaping appeared to operate differently across the settings. For example, Bogotá’s participants believed most peers are smokers (participants suggested 10–80% of their peers smoked or vaped). By comparison, Northern Irish participants estimated that a smaller proportion of their peers are smokers (participants suggested 2–30% of their peers smoked or vaped). It is important to note that in Northern Ireland, there was a more prevalent perception among participants that using e-cigarettes is more socially acceptable than combustible cigarettes, as illustrated by the following quote: “They [peers] just think it’s more appropriate, because most people think it’s scientifically proven that it doesn’t kill you as much […] Loads of people our age have them so it just seems normal, other than cigarettes, no one smokes them as much as e-cigarettes”. (ASSIST pupil, 21st May 2019, Northern Ireland).

Furthermore, in Northern Ireland, peer influence was commonly related to the need to belong and the fear of negative evaluation. For example, a pupil said: “Because if everyone in your friend group doesn’t like smoking, you won’t want to lose them as friends, especially if they’re really close and they’re good friends you don’t want to lose them if you start smoking” (Dead Cool pupil, 7th June 2019, Northern Ireland). In Bogotá, social influence was commonly related to a combination of the need to belong, the fear of negative evaluation, and social pressure from classmates and friends. For example, a participant explained: “If he is a close friend, you can say ‘no’ and he would feel you; but there are other friends that push and push. And they say ‘chicken, cry baby’, they don’t talk to you again, and you feel excluded. That is the reason why you do it.” (ASSIST pupil, 22nd October 2019, Bogotá).

### Adolescents Have Very Different Environmental Contexts and Resources

Participants identified specific contextual resources that were involved in behavioral reasoning to smoke or vape including families, schools, neighborhood, availability to purchase tobacco products, and exposure to advertising. Overall, Bogotá’s participants identified susceptibility factors related to smoking or vaping that included social pressure, smokers in families, exposure to advertising on social media, availability of purchasing cigarettes, and consumption of other substances in the neighborhood. They also identified anti-tobacco attitudes within the family as a protective factor that influenced adolescent smoking. Northern Irish participants identified susceptibility factors related to smoking or vaping that include social influence, exposure to second-hand smoke in public spaces, availability of purchasing e-cigarettes online, and social acceptability of e-cigarettes. They also identified previous anti-smoking information as a protective factor that influenced adolescent smoking. Following, we detail the contextual resources of each setting.

#### Contextual Resources in Bogotá

Pupils identified greater availability of cigarettes through family members, peer smokers at school, and unsafe neighborhoods. Adolescents also reported being exposed to smokers and tobacco products in public spaces such as streets and parks, and they could directly purchase cigarettes in the neighborhood. A pupil explained high access to tobacco products and social acceptance in the quote: “Well, they [the sellers] do not mind selling cigarettes to minors. For example, my family ask me to buy the Gold Seal [a cheap illicit brand] cigarettes, and the sellers do not tell me anything. − Where do you buy cigarettes most often? − Here in front of the school.” (ASSIST pupils, 28th May 2019, Bogotá). In the case of e-cigarettes, participants reported that they could access them through social media “giveaways,” or in their homes via other family members or older peers at school.

A further finding in Bogotá was that pupils mentioned that adolescent cigarette consumption in their schools and neighborhoods can be related to the use of other substances in risky contexts. For instance, pupils said: “ − Where are you usually exposed to secondhand smoke? − Outside of the school, when you leave it in the afternoon there are people smoking cigarettes and marihuana […] What they [the consumers] do the most is use marihuana, but also cigarettes, tobacco… Or then glue-sniffing.” (ASSIST pupils, 28^th^ May 2019, Bogotá). This quote displays that Bogotá’s pupils related smoking behavior with exposure to tobacco and use of other substances in their environment.

#### Contextual Resources in Northern Ireland

The participants outlined availability of cigarettes, through identification of smokers within families and exposure to second-hand smoke in public places such as leisure centers and neighborhoods. However, participants in Northern Ireland had a greater level of protective resources, including prior smoking information from school and greater exposure to anti-smoking advocacy through community groups. For instance, a participant said: “In my youth club one of the youth workers brought in one of the wee things that are in cigarettes, and there were loads of oil and poisonous stuff in them. − Where did you learn that? − . At youth club.” (ASSIST pupil, 5th July 2019, Northern Ireland). This quote demonstrates that the Northern Irish pupils were exposed to anti-tobacco messages in their local environments.

Participants could get access to cigarettes through adults or older peers who purchased the cigarettes for them. For example, a pupil said “Obviously I can’t just walk into a shop and just go [say] ‘can I have a 12 pack of cigarettes?’ But they’ve got fag [cigarette] houses where people buy fake cigarettes and sell them for like £4 to people, and then the same with e-cigarettes, if someone, like an adult sells it to a child and then the child will sell it to someone else” (ASSIST peer supporter, 21st May 2019, Northern Ireland). Pupils also outlined that they could purchase e-cigarettes online, and mentioned exposure to e-cigarette advertising on social media platforms.

### Perceived Behavior Change Outcomes Were Different

**Fig. 1:**
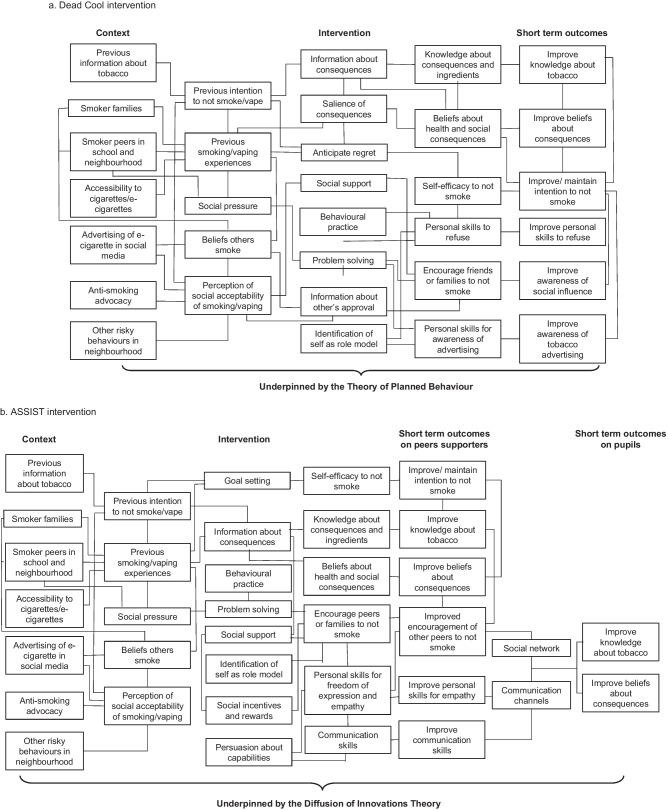
Representation of the theoretical pathways for interventions identified in focus groups in Bogotá and Northern Ireland. Based on logic models published in Hunter et al. [22]. **a** Dead Cool intervention. **b** ASSIST intervention

Both interventions had a long-term aim to prevent smoking onset among pupils, but the short-term outcomes varied according to the logic model based on the theory perspective of each intervention [22]. Categories reported in this study are graphically represented within the updated logic models of both interventions in order to display the pathways that where activated, based on the results of this study (Fig. 1).

#### Expected Outcomes of the Interventions

Expected outcomes of ASSIST include peer supporters increasing their knowledge about tobacco and health consequences, reducing their smoking intentions, and approaching classmates to communicate information about the risks of tobacco use. Changes in knowledge and attitudes towards tobacco use were expected among ASSIST pupils (non-peer supporters). In addition, increased knowledge about tobacco and health consequences, increased awareness of social influences, skills and social support, and a reduction in smoking intentions was expected among Dead Cool pupils. In 22 out of 27 focus groups, participants identified at least one specific short-term behavioral change as a result of the interventions.

Participants perceived changes related to intended outcomes were identified after the interventions. Participants mentioned improved knowledge and beliefs about the health consequences of smoking irrespective of intervention and setting. In addition, ASSIST peer supporters increased their encouragement of other peers to not smoke, which is illustrated by this quote: “I felt good because we did help some smoker classmates, and if they didn’t smoke, told them not to do it. I told them all the things that a cigarette contains” (ASSIST peer supporter, 24th October 2019, Bogotá). Meanwhile, Dead Cool pupils improved their personal skills to refuse tobacco products, as this quote illustrates: “There were scenarios where you rejected the smoking, and ways to say no without hurting their feelings and stuff” (Dead Cool pupil, 25th June 2019, Northern Ireland). This suggests that intended outcomes in both interventions are experienced in both settings.

#### Spill-Over Outcomes

We found a “spill-over” effect related to families and older peers, who were indirectly encouraged to not smoke by the participants. In Bogotá one pupil said: “I tried to convince my older brother because he has children. I said to him ‘why do you smoke? You are ending your life and you won’t see them grow’” (Dead Cool pupil, 1st October 2019, Bogotá). In Northern Ireland, participants also related their experience of trying to deter family members from smoking: “I tried to get him [the father] to stop smoking but he’ll not stop smoking anymore. I just tell them to stop, because it’s like a waste of money” (Dead Cool pupils, 7^th^ June 2019, Northern Ireland). These quotes illustrate that pupils used the resources gained from the interventions, including knowledge and beliefs about consequences, to spread social norms against tobacco within their social context.

#### Outcomes Related to Contextual Differences

Contextual differences were related to behavioral intentions to not smoke or vape and to the development of personal skills. It is important to note that general intentions to not smoke or vape were more frequently mentioned in Northern Ireland than in Bogotá, suggesting variation in behavior and intentions across the two contexts. Regarding changing intentions to not smoke after the interventions, in Bogotá, participants gave many examples of how the interventions increased their knowledge and perception of the consequences of smoking, causing a change to their intentions to smoke or vape. For example: “It was a change for me because before the program I had the thought that as soon as I turn 18, I was going to smoke my first cigarette. But, after finding out that the cigarette has consequences, well I don’t want to do it” (Dead Cool pupil, 1st October 2019, Bogotá). By comparison, participants in Northern Ireland explained how the interventions re-affirmed their intentions to not smoke through increasing their knowledge about the consequences of smoking. For example: “I wasn’t planning on smoking anyway […] It’s just been the same, because I’ve already been told not to do it. It hasn’t really changed anything, just kind of told me how bad it actually is” (ASSIST pupil, 21st May 2019, Northern Ireland). This suggested the intended outcomes of the interventions could be performed at different levels according to students’ previous contextual and personal resources.

A further difference across settings was found regarding the improved skills among participants. In Bogotá, we identified an increase in personal skills for empathy and communication skills. For example: “I learned to listen better, to have a fluent conversation, to listen to my classmates who wanted change” (ASSIST peer supporter, 22nd October 2019, Bogotá). Meanwhile, we identified increasing awareness of social influences for pupils in Northern Ireland. For example: “I learned that some teenagers think it’s cool to smoke and think that loads of other people are doing it, even if there is only 4% of people who do smoke” (Dead Cool pupil, 25th June 2019, Northern Ireland). This suggested the perceived short-term outcomes of interventions are also related to interpersonal level behavior changes.

## Discussion

This study compared the influences on behavioral change related to smoking of two prevention interventions in two different settings using a theory-driven qualitative approach. We used the TDF to explore the behavior change rationale of the evaluated smoking prevention interventions, facilitating a comprehensive theory-informed approach to uncovering the relationships between individual agency and contextual resources involved in health interventions [29]. Distinct behavioral domains were identified and updated in the logic model for each intervention, which affected participants’ reasoning across interventions and settings, including knowledge, skills, social roles for health promotion, beliefs about capabilities and consequences, intentions to smoke or vape, and social influences.

This study explored the pathways through which the smoking prevention interventions worked in settings with different resources as data were focused on participant-centered experiences of the interventions, conditions, and their outcomes [34]. The results help to unravel the complexity of how smoking prevention interventions operate in schools by shedding light on the individual, social, and contextual factors that may affect the design, implementation, scale-up, spread, and sustainability of interventions [45, 46].

In both cultural contexts and interventions, favorable or supportive behavioral resources for smoking prevention include improving participants’ beliefs and knowledge about the consequences of smoking. Our qualitative findings allowed us to demonstrate that both interventions improved personal skills. Furthermore, social norms and peer influence were related to smoking in both settings. These findings are consistent with the content of the programs and previous studies on smoking interventions in developing countries [16]. The qualitative findings also complement findings from network-based analysis in the MECHANISMS study, which likewise supported using social influence strategies in interventions to promote smoking behavioral change [6].

Our findings reinforce the notion that cross-contextual differences have implications for the implementation of behavior change programs. Our results suggest that environmental protective factors, such as being exposed to previous information about tobacco from schools, favor the intervention outcomes related to social norms changes. In environments producing high susceptibility for smoking, intervention outcomes related to social norms could be constrained in the short term by factors such as higher levels of access to tobacco products in neighborhoods. However, even in these contexts, interventions provide basic personal skills related to individual agency that can favor smoking prevention outcomes. Previous literature has identified that environmental interventions could have a beneficial impact on adolescent smoking prevention and complement school-based interventions, by increasing efforts to implement and maintain school tobacco policies [17], promoting family support [47], and implementing tobacco control policies at the local level [48].

In particular, this study provided evidence on three environmental factors that could influence the behavioral change for pupils during school-based smoking prevention interventions. First, in both settings, adolescents had previous curiosity-driven experiences of smoking and vaping, and were highly exposed to e-cigarette advertising on social media platforms. Recent literature has shown that young people are highly susceptible and widely exposed to e-cigarette marketing through the media, often targeted at specific demographic groups [49–51]. These findings highlight the importance of strengthening the implementation of tobacco control measures directed at advertising, promotion, and sponsorship through social media [12, 51, 52], and further to include e-cigarettes as tobacco products on the local regulations. There is some evidence that adolescents may be more at risk of initiating smoking following e-cigarette use [53, 54] as they are more socially acceptable than conventional cigarettes [55]. Smoking prevention strategies for adolescents should include information about risks and marketing strategies for e-cigarettes. Future researchers should also consider the role of social media in influencing adolescents’ health behavior.

Second, Bogotá’s participants are exposed to tobacco products and misuse of other substances in the neighborhood and in schools. This issue draws attention to the need for local prevention programs due to the previously reported risk of nicotine products being a gateway to misuse of other substances [1, 56]. Siquiera and Brook estimated that among Colombian adolescents, tobacco smokers had a two- to threefold higher risk of problem drug use [57]. This highlights the urgent need to implement context-based health education strategies for preventing the onset and co-use of tobacco and other substances [1, 58].

Third, in Northern Ireland, youth clubs were an important contextual resource and source of information for prevention of smoking and vaping. Previous literature has shown that youth clubs, especially (but not limited to) those focused on sports, offer many opportunities for health-promoting activities because they create a supportive environment, can strengthen community action, and can facilitate the development of personal skills [59]. The findings suggest that researchers and interventionists could learn from the resources used to promote health-enhancing behaviors in other contexts. Settings with a higher proportion of adolescents living in deprived or disadvantaged communities could benefit from strategies that target similar informal adolescent smoking prevention. This would be more relevant for LMICs where the tobacco industry has focused its marketing on youth [60] and the prevalence of adolescent smoking remains high [53].

Qualitative inquiry offers a narrative richness about behavior change interventions and their mechanisms. However, we acknowledge that single qualitative or quantitative methods [61] have limitations to (i) identify the diversity of influences on behavior change across a population of pupils; (ii) isolate the influences that may be more important for certain populations; or (iii) demonstrate that synergistic effects of the interventions’ components may induce emergent behavioral phenomena expected in a complex social system [62, 63]. As the findings of new quantitative analysis have become available for the MECHANISIMS study, we considered two main strengths of our qualitative findings. First, this study allowed a further refinement of the theory and logic model behind these interventions and the generative mechanisms by which they trigger smoking prevention [34]. Our methodological design provides comparative data on a wide range of contextual circumstances, which are likely to impact the implementation and outcomes of complex interventions [64]. Second, this study provides a basis for mixed-methods triangulation. Integrating qualitative and quantitative data can highlight areas of dissonance or complementarity that can generate insights into complex behavior change phenomena, such as adolescent smoking [30, 65]. As a consequence, future qualitative studies might explore more deeply the participant-centered experiences of interventions, conditions, and consequences, as well inquiring about participants’ theories on how interventions work [34]. Additionally, future lines of inquiry into school-based smoking prevention might usefully adopt a complexity perspective and harness suitably adapted mixed methods [66–68].

A limitation of this study is that focus groups were conducted post-intervention only, whereas a longitudinal qualitative design might more adequately capture behavioral changes over time [69]. Additionally, future qualitative studies aiming to uncover intervention mechanisms in complex behavior change interventions should include data collected from additional roles such as teachers, trainers, families, and stakeholders. This would allow a wider approach to identifying other policy, structural, and temporal contextual factors that could affect the implementation of interventions and their outcomes in practice [70].

In addition, this paper nested vaping behavior into some sub-categories of smoking behavior according to the data because intervention activities delivered mixed information about combustible cigarettes and e-cigarettes. However, our findings suggest that smoking and vaping can have different influences according to contextual factors. Future researchers should inquire about influences for smoking and vaping separately, highlighting the role of social influence and exposure to tobacco advertising.

## Conclusion

Our findings outline how individual and contextual resources influence behavioral change for school-based smoking prevention in practice. Local contextual factors and social influences affecting pupils should be considered in the implementation of interventions to prevent smoking. Using a theory-based perspective, this work contributes towards understanding how and why school-based smoking prevention interventions work, as well as offering the potential to help future researchers optimize their implementation, according to their context, to generate the targeted health outcomes.

## Supplementary Information

Below is the link to the electronic supplementary material.

Supplementary file1 (DOCX 66 KB)

## Data Availability

The aggregated datasets are available from the corresponding author. Individual data would be require IRB approval.

## Declarations

### Welfare of Animals

This paper does not contain any studies with animals performed by any of the authors.

## References

[1] U.S. Department of Health and Human Services. Preventing Tobacco Use Among Youth and Young Adults A Report of the Surgeon General. Centers for Disease Control and Prevention, National Center for Chronic Disease Prevention and Health Promotion; 2012. 30–189 p. 22876391

[2] World Health Organization. WHO global report on trends in prevalence of tobacco use 2020–2025, third edit. Third edit. World Health Organisation. Geneva, Suiza; 2019.

[3] Bonnie RJ, Stratton K, Kwan LY. Public health implications of raising the minimum age of legal access to tobacco products. Public Health Implications of Raising the Minimum Age of Legal Access to Tobacco Products. 2015. 1–378 p. 26269869

[4] Prinstein MJ, Brechwald WA, Cohen GL. Susceptibility to Peer Influence: Using a Performance-Based Measure to Identify Adolescent Males at Heightened Risk for Deviant Peer Socialization. Dev Psychol. 2011;47(4):1167–72. 21463036 10.1037/a0023274 PMC3348704

[5] Blakemore SJ, Mills KL. Is adolescence a sensitive period for sociocultural processing? Annu Rev Psychol. 2014;65. 10.1146/annurev-psych-010213-115202 24016274

[6] Montes F, Blanco M, Useche AF, Sanchez-Franco S, Caro C, Tong L, et al. Exploring the mechanistic pathways of how social network influences social norms in adolescent smoking prevention interventions. Sci Rep. 2023 Dec 1;13(1). 10.1038/s41598-023-28161-7 PMC9944961 36810585

[7] Tate C, Kumar R, Murray JM, Sanchez-Franco S, Montgomery SC, Montes F, et al. Socio-environmental and psychosocial predictors of smoking susceptibility among adolescents with contrasting socio-cultural characteristics: a comparative analysis. BMC Public Health. 2021 Dec 1;21(1). 10.1186/s12889-021-12351-x PMC8662882 34886840

[8] Kenney A, Dennis CB. Environmental paths that inform adolescent substance use prevention. J Hum Behav Soc Environ. 2019;29(7):897–908.

[9] World Health Organization. WHO report on the global tobacco epidemic 2021: addressing new and emerging products [Internet]. 2021 [cited 2022 Aug 17]. Available from: https://www.who.int/publications/i/item/9789240032095.

[10] Kyriakos CN, Zatoński MZ, Filippidis FT. Flavour capsule cigarette use and perceptions: a systematic review. Tob Control. 2023;32(e1):e83-94. 34607888 10.1136/tobaccocontrol-2021-056837 PMC10086486

[11] Zare S, Nemati M, Zheng Y. A systematic review of consumer preference for e-cigarette attributes: Flavor, nicotine strength, and type. PLoS ONE. 2018;13(3):1–19. 10.1371/journal.pone.0194145 PMC5854347 29543907

[12] Chen Y, Fowler CH, Papa VB, Lepping RJ, Brucks MG, Fox AT, et al. Adolescents’ behavioral and neural responses to e-cigarette advertising. Addict Biol. 2018;23(2):761–71. 28401670 10.1111/adb.12510 PMC5636647

[13] Badham J, Kee F, Hunter RF. Network structure influence on simulated network interventions for behaviour change. Soc Networks [Internet]. 2021;64:55–62. Available from: https://www.sciencedirect.com/science/article/pii/S037887332030071X.

[14] Macarthur G, Caldwell DM, Redmore J, Watkins SH, Kipping R, White J, et al. Individual-, family-, and school-level interventions targeting multiple risk behaviours in young people. Cochrane Database of Systematic Reviews. 2018;2018(10). 10.1002/14651858.CD009927.pub2 PMC6517301 30288738

[15] de Kleijn MJJ, Farmer MM, Booth M, Motala A, Smith A, Sherman S, et al. Systematic review of school-based interventions to prevent smoking for girls. Syst Rev. 2015;4(1):1–12. 26272326 10.1186/s13643-015-0082-7 PMC4536766

[16] Huriah T, Lestari VD. School-based smoking prevention in adolescents in developing countries: A literature review. Open Access Maced J Med Sci. 2020;8:84–9.

[17] Mélard N, Grard A, Robert PO, Kuipers MAG, Schreuders M, Rimpelä AH, et al. School tobacco policies and adolescent smoking in six European cities in 2013 and 2016: A school-level longitudinal study. Prev Med (Baltim). 2020;1:138. 10.1016/j.ypmed.2020.106142 32450162

[18] Skivington K, Matthews L, Simpson SA, Craig P, Baird J, Blazeby JM, et al. Framework for the development and evaluation of complex interventions: Gap analysis, workshop and consultation-informed update. Health Technol Assess (Rockv). 2021;25(57):i–132. 10.3310/hta25570 PMC7614019 34590577

[19] Astbury B, Leeuw FL. Unpacking Black Boxes: Mechanisms and Theory Building in Evaluation. Am J Eval. 2010;31(3):363–81.

[20] Long E, Valente TW. Perceived Social Acceptability and Longitudinal Trends in Adolescent Cigarette Smoking. Prev Sci. 2019;20(6):824–32. 30168036 10.1007/s11121-018-0945-y

[21] Montgomery SC, Donnelly M, Bhatnagar P, Carlin A, Kee F, Hunter RF. Peer social network processes and adolescent health behaviors: A systematic review. Prev Med (Baltim). 2020;130(November 2019):105900. 10.1016/j.ypmed.2019.105900 31733224

[22] Hunter RF, Montes F, Murray JM, Sanchez-Franco SC, Montgomery SC, Jaramillo J, et al. MECHANISMS Study: Using Game Theory to Assess the Effects of Social Norms and Social Networks on Adolescent Smoking in Schools—Study Protocol. Front Public Health. 2020;8(August):1–14. 32850598 10.3389/fpubh.2020.00377 PMC7417659

[23] Thurston A, Dunne L, Kee F, Gildea A, Craig N, Stark P, et al. A randomized controlled efficacy trial of a smoking prevention programme with Grade 8 students in high schools. Int J Educ Res. 2019;2019(93):23–32.

[24] Campbell R, Starkey F, Holliday J, Audrey S, Bloor M, Parry-Langdon N, et al. An informal school-based peer-led intervention for smoking prevention in adolescence (ASSIST): a cluster randomised trial. The Lancet. 2008;371(9624):1595–602. 10.1016/S0140-6736(08)60692-3 PMC2387195 18468543

[25] Dalkin SM, Greenhalgh J, Jones D, Cunningham B, Lhussier M. What’s in a mechanism? Development of a key concept in realist evaluation. Implement Sci. 2015;10(1):1–7. 25885787 10.1186/s13012-015-0237-x PMC4408605

[26] Rogers EM. Diffusion of innovations. 5th ed. New York: Free Press; 2003.

[27] Ajzen I. The Theory of Planned Behavior. Organ Behav Hum Decis Process. 1991;50:179–211.

[28] Atkins L, Francis J, Islam R, O’Connor D, Patey A, Ivers N, et al. A guide to using the Theoretical Domains Framework of behaviour change to investigate implementation problems. Implement Sci. 2017;12(1):1–18. 28637486 10.1186/s13012-017-0605-9 PMC5480145

[29] McGowan LJ, Powell R, French DP. How can use of the Theoretical Domains Framework be optimized in qualitative research? A rapid systematic review. Br J Health Psychol. 2020;25(3):677–94. 32558289 10.1111/bjhp.12437

[30] Johnson RB, Russo F, Schoonenboom J. Causation in Mixed Methods Research: The Meeting of Philosophy, Science, and Practice. J Mix Methods Res. 2019;13(2):143–62.

[31] Shochet I, Montague R, Smith C, Dadds M. A qualitative investigation of adolescents’ perceived mechanisms of change from a universal school-based depression prevention program. Int J Environ Res Public Health. 2014;11(5):5541–54. 24859679 10.3390/ijerph110505541 PMC4053869

[32] McMahon NE, Visram S, Connell LA. Mechanisms of change of a novel weight loss programme provided by a third sector organisation: A qualitative interview study. BMC Public Health [Internet]. 2016;16(1):1–11. Available from: 10.1186/s12889-016-3063-4. 10.1186/s12889-016-3063-4 PMC4862220 27165634

[33] Myneni S, Cobb N, Cohen T. In pursuit of theoretical ground in behavior change support systems: Analysis of peer-to-peer communication in a health-related online community. J Med Internet Res. 2016;18(2). 10.2196/jmir.4671 PMC4756252 26839162

[34] Bonell C, Warren E, Melendez-Torres GJ. Methodological reflections on using qualitative research to explore the causal mechanisms of complex health interventions. Evaluation. 2022;28(2):166–81.

[35] The World Bank. World Development Indicators [Internet]. 2021. Available from: http://wdi.worldbank.org/table. Date

[36] The World Bank. The World Bank, Data, Colombia. 2019.

[37] Mary Assunta. Global Tobacco Industry Interference Index 2021 [Internet]. Bangkok; 2021 Nov. Available from: www.globaltobaccoindex.org.

[38] Ministerio de Salud y Protección Social. Encuesta Nacional De Tabaquismo En Jóvenes De Colombia (Entj). 2020. 140 p.

[39] Northern Ireland Statistics and Research Agency. Young Persons’ Behaviour and Attitudes Survey Key Findings 2019–2020. 2020.

[40] Cancer Focus Northern Ireland. Smokebusters [Internet]. Cancer Focus Northern Ireland. 2021 [cited 2021 May 30]. Available from: https://cancerfocusni.org/primary-programmes/smokebusters/.

[41] Ministerio de Salud y Protección Social. Estrategia de entorno educativo saludable. Ministerio de salud y proteccion social 2018 p. 1–37.

[42] Sánchez-Franco S, Arias LF, Jaramillo J, Murray JM, Hunter RF, Llorente B, et al. Cultural adaptation of two school-based smoking prevention programs in Bogotá , Colombia. Transl Behav Med. 2021;(April). 10.1093/tbm/ibab019 PMC8499713 33899915

[43] Tate C, Kumar R, Murray JM, Sanchez-Franco S, Montgomery SC, Montes F, et al. Socio-environmental and psychosocial predictors of smoking susceptibility among adolescents with contrasting socio-cultural characteristics: a comparative analysis. BMC Public Health [Internet]. 2021;21(1):1–12. Available from: 10.1186/s12889-021-12351-x. 10.1186/s12889-021-12351-x PMC8662882 34886840

[44] Elo S, Kyngäs H. The qualitative content analysis process. J Adv Nurs [Internet]. 2008 Apr 1;62(1):107–15. Available from: 10.1111/j.1365-2648.2007.04569.x. 10.1111/j.1365-2648.2007.04569.x 18352969

[45] Vanderkruik R, McPherson ME. A Contextual Factors Framework to Inform Implementation and Evaluation of Public Health Initiatives. Am J Eval. 2017;38(3):348–59.

[46] Shaw J, Gray CS, Baker GR, Denis JL, Breton M, Gutberg J, et al. Mechanisms, contexts and points of contention: Operationalizing realist-informed research for complex health interventions. BMC Med Res Methodol. 2018;18(1):1–12. 30587138 10.1186/s12874-018-0641-4 PMC6307288

[47] Zaborskis A, Kavaliauskienė A, Eriksson C, Klemera E, Dimitrova E, Melkumova M, et al. Family support as smoking prevention during transition from early to late adolescence: a study in 42 countries. Int J Environ Res Public Health. 2021 Dec 1;18(23). 10.3390/ijerph182312739 PMC8656923 34886464

[48] Prado-Galbarro FJ, Auchincloss AH, Pérez-Ferrer C, Sanchez-Franco S, Barrientos-Gutierrez T. Adolescent tobacco exposure in 31 latin american cities before and after the framework convention for tobacco control. Int J Environ Res Public Health. 2020;17(20):1–15. 10.3390/ijerph17207423 PMC7601699 33053821

[49] Lee SJ, Rees VW, Yossefy N, Emmons KM, Tan ASL. Youth and Young Adult Use of Pod-Based Electronic Cigarettes from 2015 to 2019: A Systematic Review. JAMA Pediatr. 2020;174(7):714–20. 32478809 10.1001/jamapediatrics.2020.0259 PMC7863733

[50] Virgili F, Nenna R, Ben David S, Mancino E, Di Mattia G, Matera L, et al. E-cigarettes and youth: an unresolved Public Health concern. Ital J Pediatr [Internet]. 2022 Dec [cited 2022 Aug 17];48(1). Available from: https://pubmed.ncbi.nlm.nih.gov/35701844/. 10.1186/s13052-022-01286-7 PMC9194784 35701844

[51] Emery SL, Vera L, Huang J, Szczypka G. Wanna know about vaping? Patterns of message exposure, seeking and sharing information about e-cigarettes across media platforms. Tob Control. 2014;23:iii17–25. 10.1136/tobaccocontrol-2014-051648 PMC4078680 24935893

[52] Ford A, MacKintosh AM, Bauld L, Moodie C, Hastings G. Adolescents’ responses to the promotion and flavouring of e-cigarettes. Int J Public Health. 2016;61(2):215–24. 26650455 10.1007/s00038-015-0769-5 PMC4819499

[53] Drope J, Schluger N, Cahn Z, Drope J, Hamill S, Islami F, et al. The Tobacco Atlas. Atlanta: American Cancer Society and Vital Strategies. the American Cancer Society, Inc.; 2018. p. 26.

[54] Kowitt SD, Goldstein AO, Sutfin EL, Osman A, Meernik C, Heck C, et al. Adolescents’ first tobacco products: Associations with current multiple tobacco product use. PLoS ONE. 2019;14(5):1–16. 10.1371/journal.pone.0217244 PMC6532893 31120972

[55] Gorukanti A, Delucchi K, Ling P, Fisher-Travis R, Halpern-Felsher B. Adolescents’ attitudes towards e-cigarette ingredients, safety, addictive properties, social norms, and regulation. Prev Med (Baltim). 2017;94:65–71. 10.1016/j.ypmed.2016.10.019 PMC5373091 27773711

[56] Rajabi A, Dehghani M, Shojaei A, Farjam M, Motevalian SA. Association between tobacco smoking and opioid use: A meta-analysis. Addict Behav. 2018;2019(92):225–35. 10.1016/j.addbeh.2018.11.043 30685521

[57] Siqueira LM, Brook JS. Tobacco use as a predictor of illicit drug use and drug-related problems in Colombian youth. J Adolesc Health. 2003;32(1):50–7. 12507801 10.1016/s1054-139x(02)00534-7

[58] Ramo DE, Liu H, Prochaska JJ. Tobacco and marijuana use among adolescents and young adults: A systematic review of their co-use. Clin Psychol Rev. 2012;32:105–21. 22245559 10.1016/j.cpr.2011.12.002 PMC3267894

[59] Geidne S, Quennerstedt M, Eriksson C. The youth sports club as a health-promoting setting: An integrative review of research. Scand J Public Health. 2013;41:269–83. 23349167 10.1177/1403494812473204 PMC3807854

[60] Gilmore A, Fooks G, Drope J, Bialous SA, Jackson R. Exposing and addressing tobacco industry conduct in low and middle income countries. Lancet. 2015;385(9972):1029–43. 25784350 10.1016/S0140-6736(15)60312-9 PMC4382920

[61] VanderWeele TJ. Mediation and mechanism. Eur J Epidemiol. 2009;24:217–24. 19330454 10.1007/s10654-009-9331-1

[62] Harris FM, Maxwell M, O’Connor R, Coyne JC, Arensman E, Coffey C, et al. Exploring synergistic interactions and catalysts in complex interventions: Longitudinal, mixed methods case studies of an optimised multi-level suicide prevention intervention in four european countries (Ospi-Europe). BMC Public Health. 2016;16(1):1–9. 26979461 10.1186/s12889-016-2942-z PMC4791791

[63] Moore GF, Evans RE, Hawkins J, Littlecott H, Melendez-Torres GJ, Bonell C, et al. From complex social interventions to interventions in complex social systems: Future directions and unresolved questions for intervention development and evaluation. Evaluation. 2018;00:1–23. 10.1177/1356389018803219 PMC6330692 30705608

[64] Manzano A. Conducting focus groups in realist evaluation. Evaluation. 2022 Oct 1. 10.1177/13563890221124637 PMC9530522 36212730

[65] Tonkin-Crine S, Anthierens S, Hood K, Yardley L, Cals JWL, Francis NA, et al. Discrepancies between qualitative and quantitative evaluation of randomised controlled trial results: Achieving clarity through mixed methods triangulation. Implement Sci. 2016;11(1):1–8. 27175799 10.1186/s13012-016-0436-0 PMC4866290

[66] Kallemeyn LM, Hall JN, Gates E. Exploring the Relevance of Complexity Theory for Mixed Methods Research. J Mix Methods Res. 2019;1–17.

[67] Gear C, Eppel E, Koziol-Mclain J. Advancing Complexity Theory as a Qualitative Research Methodology. Int J Qual Methods. 2018;17:1–10.

[68] Bilodeau A, Potvin L. Unpacking complexity in public health interventions with the Actor-Network Theory. Health Promot Int. 2016;1–9. 10.1093/heapro/daw062 27492825

[69] Corepal R, Best P, O’Neill R, Tully MA, Edwards M, Jago R, et al. Exploring the use of a gamified intervention for encouraging physical activity in adolescents: A qualitative longitudinal study in Northern Ireland. BMJ Open. 2018;8: e019663. 29678971 10.1136/bmjopen-2017-019663 PMC5914771

[70] Sheaff R, Doran N, Harris M, Lang I, Medina-Lara A, Fornasiero M, et al. Categories of context in realist evaluation. Evaluation. 2021;27(2):184–209.

