# Supplementary material for: How Do Adolescent Smoking Prevention Interventions Work in Different Contextual Settings? A Qualitative Comparative Study Between the UK and Colombia
Source: Int J Behav Med. 2023 Sep 11;31(5):691–704. doi: 10.1007/s12529-023-10211-z (PMC11452532; doi:10.1007/s12529-023-10211-z)
Supplement: Supplementary file 1 — Supplementary file1 (DOCX 66 KB) [file 12529_2023_10211_MOESM1_ESM.docx]

**Electronic Supplementary Material 1 – COREQ checklist (Tong et al., 2007)**

The data reported in this checklist correspond to the qualitative component of the MECHANISMS study. The study aims to compare the influences on the ASSIST and Dead Cool participants’ behavioral change related to smoking or vaping in two different contexts

(Bogotá and Northern Ireland). The A Stop Smoking in Schools Trial (ASSIST) program harnesses peer inﬂuence for spreading anti-smoking messages, whilst the Dead Cool program invokes a more conventional classroom pedagogy approach. Both interventions were initially developed in the UK but were culturally adapted to be implemented in a Colombian setting.

| **Domain 1: Research team and reflexivity** | |
| --- | --- |
| **Personal characteristics** | |
| 1. Interviewer/facilitator  *Which author/s conducted the interview or focus group?* | SSF in Bogotá; SCM in Northern Ireland |
| 2. Credentials  *What were the researcher’s credentials? e.g., PhD,*  *MD* | SSF: MPH from Universidad de los Andes  SCM: PhD BSc from Queen’s University Belfast |
| 3. Occupation  *What was their occupation at the time of the study?* | SSF: Professional researcher at Universidad de Los Andes  SCM: Research Assistant at Queen’s University Belfast |
| 4. Gender  *Was the researcher male or female?* | Both researchers were female |
| 5. Experience and training  *What experience or training did the researcher have?* | SSF is a psychologist with 5 years of experience as a professional researcher, and experience on the design, management and evaluation of community projects in educational contexts with children and adolescents.  SCM is a postdoctoral researcher with prior experience working with adolescent health behavior change in school settings. SCM holds a PhD in Public Health Medicine, during which she undertook training in leading adolescent focus groups and conducting qualitative analysis. |
| **Relationship with participants** | |
| 6. Relationship established  *Was a relationship established prior to study commencement?* | No |
| 7. Participant knowledge of the interviewer  *What did the participants know about the researcher? e.g., personal goals, reasons for doing the research* | None |
| 8. Interviewer characteristics  *What characteristics were reported about the interviewer/facilitator? e.g. Bias, assumptions, reasons and interests in the research topic* | No characteristics were reported |
| **Domain 2: Study design** | |
| **Theoretical framework** | |
| 9. Methodological orientation and Theory  *What methodological orientation was stated to underpin the study? e.g., grounded theory, discourse analysis, ethnography, phenomenology, content analysis* | Deductive content analysis |
| **Participant selection** | |
| 10. Sampling  *How were participants selected?* | Stratified sampling: The research team looked for the same number of boys and girls with different characteristics that form a representative and unbiased sample in the participating classrooms.  Participants were in both settings and bot programs. |
| 11. Method of approach  *How were participants approached? e.g., face-to-face, telephone, mail, email* | Twelve schools participated at this stage of the MECHANISMS study. Six schools were included in the ASSIST intervention (3 in Northern Ireland and 3 in Bogotá) and six schools were included in the Dead Cool intervention (3 in Northern Ireland and 3 in Bogotá).  In Bogotá, focus group participants were selected and approached face-to-face by the research team.  In NI, participants were informed about the focus group study component during data collection study visits and provided with study information sheets. Participant consent was obtained from interested students who were willing to take part, and focus group participants were selected at random. |
| 12. Sample size  *How many participants were in the*  *study?* | One hundred sixty-five (195) students participated in the focus groups of intervention (N=56 in Bogotá and N= 139 NI).  In Bogotá, we conducted 4 focus groups (N=24) among the Dead Cool schools and 6 focus groups (N=32) among the ASSIST schools.  In Northern Ireland, we conducted 4 focus groups (N=55) among Dead Cool schools and 9 focus groups (N=84) among ASSIST schools. |
| 13. Non-participation  *How many people refused to participate or dropped out? Reasons?* | At the time of the MECHANISIMS study, 127 students did opt-out, left schools or withdrew. During the focus group stage, no students withdrew. |
| **Setting** | |
| 14. Setting of data collection  *Where was the data collected? e.g., home, clinic, workplace* | Focus groups were held in each of the participating schools. |
| 15. Presence of non-participants  *Was anyone else present besides the participants and researchers?* | No |
| 16. Description of sample  *What are the important characteristics of the sample? e.g. demographic data, date* | Boys and girls aged 11–15 years in secondary schools (i.e., post-primary, 7^th^ grade in Bogotá and Year 9 in Northern Ireland). |
| **Data collection** | |
| 17. Interview guide  *Were questions, prompts, guides provided by the authors? Was it pilot tested?* | Questions and prompts where previously designed by the researchers. See the focus group guide in supplement 2. The focus group guide was translated and back translated by bilingual speakers/translators.  Yes, there was a pilot. The research team tested all the interventions during the previous stage of the study. |
| 18. Repeat interviews  *Were repeat interviews carried out? If yes, how many?* | No |
| 19. Audio/visual recording  *Did the research use audio or visual recording to collect the data?* | All focus groups were audio-recorded, then the research team transcribed the recordings. During this process, participants remained anonymous to ensure confidentiality agreements. |
| 20. Field notes  *Were field notes made during and/or after the interview or focus group?* | No |
| 21. Duration  *What was the duration of the interviews or focus group?* | Durations ranged from 26 minutes to one hour and one minute. |
| 22. Data saturation  *Was data saturation discussed?* | Yes. After the Focus Groups and during the deductive content analysis, data saturation was discussed, and the researchers agreed that data saturation had been achieved. |
| 23. Transcripts returned  *Were transcripts returned to participants for comment and/or correction?* | No |
| **Domain 3: Analysis and findings** | |
| **Data analysis** | |
| 24. Number of data coders  *How many data coders coded the data?* | Four coders in total. The first stage was coded by ETN, PGA and ALM. Then the coding consistency was co-checked by two coders (ETN and AMRV) and the lead authors (SSF and SCM). |
| 25. Description of the coding tree  *Did authors provide a description of the coding tree?* | Yes. The codebook was defined based on the Theoretical Domains Framework (TDF) |
| 26. Derivation of themes  *Were categories identified in advance or derived from the data?* | Categories were derived from the Theoretical Domains Framework (TDF). The first coding cycle served as an exploratory categorization, second coding cycle desegregated and subdivided data. |
| 27. Software  *What software, if applicable, was used to manage the data?* | NVIVO 12 Pro (QSR International Pty Ltd. Version 12 Pro, 2020). |
| 28. Participant checking  *Did participants provide feedback on the findings?* | No, there was no process of participant checking of research findings. |
| **Reporting** | |
| 29. Quotations presented  *Were participant quotations presented to illustrate the themes / findings? Was each quotation identified? e.g., participant number* | Yes, participant quotations are present to illustrate findings with each quotation identified by an anonymized participant (defined by setting, intervention, date of focus group). |
| 30. Data and findings consistent  *Was there consistency between the data presented and the findings?* | Yes, the results section shows the findings according to the codebook. A supplement is added to inform the NVivo results. |
| 31. Clarity of major themes  *Were major themes clearly presented in the findings?* | Yes, the findings draw upon the content of the hierarchical categories and sub-categories. |
| 32. Clarity of minor themes  *Were minor themes clearly presented in the findings?* | Yes, the findings draw upon the content of the hierarchical categories and sub-categories. |

**References:**

Tong, A., Sainsbury, P., & Craig, J. (2007). Consolidated criteria for reporting qualitative research (COREQ): A 32-item checklist for interviews and focus groups. *International Journal for Quality in Health Care*, *19*(6), 349–357. Doi: 10.1093/intqhc/mzm042

**Electronic Supplementary Material 2 – Sociodemographic characteristics of students that received smoking prevention interventions**

|  | **Northern Ireland** | | |  | **Bogotá** | | |  | **NI vs. Btá** |
| --- | --- | --- | --- | --- | --- | --- | --- | --- | --- |
|  | **A** | **DC** |  |  | **A** | **DC** |  |  |  |
|  | **n (%)** | **n (%)** | ***p*^c^** |  | **n (%)** | **n (%)** | ***p*^c^** |  | ***p***^d^ |
| Total | 393 | 284 |  |  | 333 | 305 |  |  |  |
| Girl/prefer not to say | 171 (47.24) | 157 (60.38) | 0.001 |  | 160 (49.08) | 150 (49.18) | 0.980 |  | 0.202 |
| Age |  |  |  |  |  |  |  |  |  |
| 11-12 years old | 139 (38.4) | 104 (40) | 0.686 |  | 121 (36.34) | 100 (32.79) | 0.019 |  | <0.001 |
| 13-14 years old | 223 (61.6) | 156 (60) |  |  | 191 (57.36) | 166 (54.43) |  |  |  |
| 15+ years old | . | . |  |  | 21 (6.31) | 39 (12.79) |  |  |  |
| Ethnic minority | 39 (10.83) | 8 (3.08) | <0.001 |  | 38 (11.66) | 50 (16.39) | 0.086 |  | <0.001 |
| Home composition |  |  |  |  |  |  |  |  | <0.001 |
| Single parents | 70 (19.44) | 37 (14.23) | 0.057 |  | 129 (39.57) | 121 (39.67) | 0.969 |  |  |
| Both parents | 279 (77.5) | 220 (84.62) |  |  | 174 (53.37) | 164 (53.77) |  |  |  |
| Other adults | 11 (3.06) | 3 (1.15) |  |  | 23 (7.06) | 20 (6.56) |  |  |  |
| Deprivation Rank^a^ | |  |  |  |  |  |  |  |  |
| Low (0-300) | 141 (43.65) | 115 (49.15) | <0.001 |  | . | . |  |  | - |
| Middle (301-600) | 69 (21.36) | 105 (44.87) |  |  | . | . |  |  |  |
| High (601-890) | 113 (34.98) | 14 (5.98) |  |  | . | . |  |  |  |
| Socioeconomic level^b^ | |  |  |  |  |  |  |  |  |
| Low (1-2) | . | . |  |  | 186 (56.71) | 151 (51.19) | 0.385 |  | - |
| Middle (3-4) | . | . |  |  | 141 (42.99) | 143 (48.47) |  |  |  |
| High (5-6) | . | . |  |  | 1 (0.3) | 1 (0.34) |  |  |  |
| Note: Sample sizes may not sum to total N due to missing data.  A: ASSIST intervention; DC: Dead Cool intervention  ^a^ Northern Ireland Multiple Deprivation Measure rank derived from NISRA data.  ^b^ Reported Colombian socioeconomic status, corresponding to an official six-level measurement that includes external characteristics of housing according to the DANE data.  ^c^ *p-value* of chi-square test for independence of dichotomized variables and interventions  ^d^ *p-value* of chi-square test for independence of dichotomized variable and setting (Northern Ireland and Bogotá) | | | | | | | | | |

**Electronic Supplementary Material 3 – Focus group guides**

**Focus Group Topic Guide - Pupils**

At the beginning of the interview, the researcher will introduce himself/herself and give a brief overview of the study. The researcher will discuss confidentiality, group rules, and consent with the participants, and allow for any questions or concerns participants may have to be aired. Participants will be reminded that they are entitled to withdraw at any stage without giving a reason. Participants will be invited to introduce themselves and there will follow a short ice-breaker conversation.

*Please note that the following is an indicative topic guide. Each of the six broad categories will be addressed, with letters indicating prompts.*

*The focus group will address:*

1. Pupils’ past/current smoking behavior.
   1. Do any of you currently smoke or use e-cigarettes?
   2. Have you ever smoked or used e-cigarettes (even just a puff or two)?
   3. If you have tried a cigarette, what were the circumstances which led to your smoking? Who were you with? Where were you? What were you doing?
2. Pupils’ attitudes towards smoking.
   1. How do you feel about smoking in general? If you are a current smoker do you intend to quit? If you have never smoked do you intend to start smoking in the future?
   2. Do you think smoking should be banned in public places, bars, restaurants, shopping malls etc.?
   3. Thinking about the potential risks and benefits of smoking, what do you think are the main **short-term** risks in terms of your health, finances, potentially becoming addicted to smoking, and your relationships with others?
   4. What are the **long-term** risks?
   5. Thinking about the potential risks and benefits of smoking, what do you think are the main **short-term** benefits in terms of your health, finances, potentially becoming addicted to smoking, and your relationships with others?
   6. Are there any **long-term** benefits?
3. Influences on smoking behavior.
   1. What do you think are the major influences that might encourage you, or other young people, to start smoking?
   2. Have you seen any advertisements for smoking in the media?
   3. Can you remember seeing examples of smoking in movies, TV shows, social media, magazines or newspapers? If so, what were they?
4. Social norms for smoking and past/current smoking behavior of family and friends.
   1. Roughly what percentage of young people of your age do you **personally** think smoke in Northern Ireland? Why do you think this?
   2. Do you think that most people of your age expect a lot of other young people to smoke?
   3. Do you **personally** think that it is ever appropriate for young people of your age to smoke?
   4. Do you think that most people of your age think it is appropriate/OK for other young people to smoke?
   5. Can you estimate how many people smoke in your year at your school?
   6. Do any of your family members smoke? If so, who?
   7. Do any of your close friends smoke? If so, how many?
   8. How often are you exposed to second-hand smoke from friends or family members? How does that make you feel?
5. Social support for smoking from family and friends.
   1. If you started smoking and your parents/family found out, how do you think they would feel and react?
   2. If you started smoking, how do you think your closest friends would react?
   3. If one of your friends were to offer you a cigarette, do you think you could refuse? How would you go about it?
   4. If one of your friends were to start smoking, do you think you could intervene to encourage them to stop smoking? How would you go about it?
6. Awareness of and thoughts about the program.
   1. Has taking part encouraged you to change your attitudes towards smoking or have you changed your smoking behavior?
   2. Was there anything that you learnt which surprised you about smoking?
   3. Is there anything that you would change if you were to take part again?
   4. How did you feel about completing the experiments and the surveys? How did you feel about using the carbon monoxide monitoring device?
   5. Are there any parts of the program which you did not enjoy? Why was this? [Dead Cool only].
   6. Would you recommend taking part to other young people your age? [Dead Cool only].
   7. Was there anything left out of the program which you felt should have been covered? Was there anything you would like to have seen more of? [Dead Cool only].
   8. In the past few weeks, can you remember having a conversation where either you or one of your friends brought up the topic of smoking and discussed the risks and benefits of smoking? How did the conversation go? What was the outcome? How many conversations can you remember? [ASSIST only].
   9. Have you seen any posters around your school encouraging you not to smoke? [ASSIST only].

**Focus Group Topic Guide – Peer supporter ASSIST**

At the beginning of the interview, the researcher will introduce himself/herself and give a brief overview of the study. The researcher will discuss confidentiality, group rules, and consent with the participants, and allow for any questions or concerns participants may have to be aired. Participants will be reminded that they are entitled to withdraw at any stage without giving a reason. Participants will be invited to introduce themselves and there will follow a short ice-breaker conversation.

*Please note that the following is an indicative topic guide. Each of the four broad categories will be addressed, with letters indicating prompts.*

*The focus group will address:*

1. Pupils’ past/current smoking behavior.
   1. Do any of you currently smoke or use e-cigarettes?
   2. Have you ever smoked or used e-cigarettes (even just a puff or two)?
   3. If you did smoke, were you encouraged to stop smoking by taking part in the peer supporter training?
2. Social norms for smoking and smoking behavior of friends.
   1. Roughly what percentage of young people of your age do you **personally** think smoke in Northern Ireland / Colombia? Why do you think this?
   2. Do you think that most people of your age expect a lot of other young people to smoke?
   3. Do you **personally** think that it is ever appropriate for young people of your age to smoke?
   4. Do you think that most people of your age think it is appropriate/OK for other young people to smoke?
   5. Do any of your close friends smoke? If so, how many?
   6. Can you estimate how many people smoke in your year at your school?
3. Experiences during peer supporter training
   1. How did you feel about the amount of time you had to spend away from your usual school activities and lessons for peer supporter training and for the follow-up sessions?
   2. Are there any parts of the training which you found particularly enjoyable?
   3. Are there parts of the training you did not enjoy? Why was this?
   4. Would you recommend taking part to other young people your age?
   5. Is there anything you would like to see more of should you take part again in the future?
   6. Where there any aspects of the training that you found too demanding?
4. Outcomes and implementation.
   1. In the past few weeks, can you remember initiating a conversation with one or more of your friends about the risks and benefits of smoking? How did the conversation go? What was the outcome? How many conversations can you remember?
   2. How did you feel approaching your friends to have a conversation about smoking? Were you nervous? Did you feel the training had prepared you well? Did this change over the course of the program as you gained experience and got more feedback from the trainers?
   3. Did you make use of the diary to record your conversations? Did you find this useful for monitoring your conversations and improving them?
   4. Did you put up any posters around your school to encourage your friends not to smoke?
   5. Has this encouraged your friends to change their attitudes towards smoking? Have any of your friends changed their smoking behavior as a result?
   6. Do you feel that you could make use of the skills you developed during the peer supporter training in your later education, career or personal life? How?

**Electronic Supplementary Material 4 – Saturations of all categories by intervention and setting**

Saturation refers to the number of times that a category was mentioned by the participants during the focus groups.

| **Domains** |  | **Total** |  | **Intervention** | |  | **Setting** | |
| --- | --- | --- | --- | --- | --- | --- | --- | --- |
|  |  |  |  | **ASSIST** | **Dead Cool** |  | **Bogotá** | **Northern Ireland** |
| **Beliefs about Consequences** |  | **254** |  | **166** | **88** |  | **71** | **183** |
| Health risk perception |  | 168 |  | 84 | 58 |  | 37 | 105 |
| Social risk perception |  | 92 |  | 64 | 28 |  | 19 | 73 |
| Benefits perception |  | 78 |  | 50 | 20 |  | 25 | 45 |
| **Social Influences** |  | **188** |  | **124** | **64** |  | **58** | **130** |
| Peer influences |  | 76 |  | 44 | 29 |  | 17 | 56 |
| Family |  | 69 |  | 38 | 27 |  | 17 | 48 |
| Descriptive social norm |  | 72 |  | 44 | 14 |  | 27 | 31 |
| Social acceptance |  | 41 |  | 26 | 13 |  | 12 | 27 |
| **Environmental context and resources** |  | **157** |  | **102** | **55** |  | **67** | **90** |
| Exposure to tobacco-related advertising |  | 51 |  | 32 | 19 |  | 18 | 33 |
| Availability of tobacco products |  | 46 |  | 26 | 17 |  | 15 | 28 |
| Family |  | 46 |  | 33 | 13 |  | 22 | 24 |
| Schools |  | 22 |  | 14 | 8 |  | 17 | 5 |
| Neighborhoods |  | 21 |  | 14 | 7 |  | 9 | 12 |
| **Knowledge** |  | **102** |  | **63** | **39** |  | **46** | **56** |
| Knowledge of cigarettes and smoking behavior |  | 121 |  | 60 | 36 |  | 40 | 56 |
| Gaps of knowledge |  | 16 |  | 10 | 6 |  | 10 | 6 |
| **Intentions** |  | **99** |  | **61** | **38** |  | **39** | **60** |
| To not smoke |  | 54 |  | 27 | 24 |  | 14 | 37 |
| To smoke |  | 42 |  | 26 | 15 |  | 23 | 18 |
| To vape |  | 20 |  | 13 | 6 |  | 12 | 7 |
| **Perceived outcomes** |  | **79** |  | **37** | **42** |  | **36** | **43** |
| Change in perception |  | 71 |  | 32 | 39 |  | 35 | 36 |
| No change in perception |  | 14 |  | 8 | 6 |  | 1 | 13 |
| **Skills** |  | **76** |  | **51** | **25** |  | **33** | **43** |
| Communication Skills |  | 43 |  | 34 | 7 |  | 24 | 17 |
| Refusing offers to smoke or vape from others |  | 35 |  | 17 | 18 |  | 9 | 26 |
| **Promotor role (social role)** |  | **65** |  | **53** | **12** |  | **24** | **41** |
| Peer supporter |  | 35 |  | 32 | 2 |  | 19 | 15 |
| Peers |  | 26 |  | 19 | 7 |  | 3 | 23 |
| Family |  | 10 |  | 7 | 3 |  | 3 | 7 |
| **Beliefs about capabilities** |  | **48** |  | **20** | **28** |  | **9** | **39** |
| Self-efficacy to refuse |  | 40 |  | 14 | 17 |  | 8 | 23 |
| Perceived competence to encourage others not to smoke |  | 21 |  | 9 | 12 |  | 2 | 19 |
